# Relationships among Constitution, Stress, and Discomfort in the First Trimester

**DOI:** 10.1155/2012/486757

**Published:** 2011-08-15

**Authors:** Hsiao-Ling Wang, Tzu-Chi Lee, Shih-Hsien Kuo, Fan-Hao Chou, Li-Li Chen, Yi-Chang Su, Lih-Mih Chen

**Affiliations:** ^1^College of Nursing, Fooyin University, 151 Jinxue Road, Daliao District, Kaohsiung 83102, Taiwan; ^2^Department of Public Health, Kaohsiung Medical University, 100 Shi-Chuan 1st Road, San Ming District, Kaohsiung 80708, Taiwan; ^3^Department of Nutrition and Health Science, College of Health and Medical Science, Fooyin University, 151 Jinxue Road, Daliao District, Kaohsiung 83102, Taiwan; ^4^College of Nursing, Kaohsiung Medical University, 100, Shi-Chuan 1st Road, San Ming District, Kaohsiung 80708, Taiwan; ^5^School of Nursing, College of Health Care, China Medical University, 91 Hsueh-Shih Road, Taichung 40402, Taiwan; ^6^College of Chinese Medicine, China Medical University, 91 Hsueh-Shih Road, Taichung 40402, Taiwan

## Abstract

The purpose of this study was to explore correlations among constitution, stress, and discomfort symptoms during the first trimester of pregnancy. We adopted a descriptive and correlational research design and collected data from 261 pregnant women during their first trimester in southern Taiwan using structured questionnaires. Results showed that (1) stress was significantly and positively correlated with Yang-Xu, Yin-Xu, and Tan-Shi-Yu-Zhi constitutions, respectively; (2) Yin-Xu and Tan-Shi-Yu-Zhi constitutions had significant correlations with all symptoms of discomfort, while Yang-Xu had significant correlations with all symptoms of discomfort except for “running nose”; (3) Tan-Shi-Yu-Zhi constitution and stress were two indicators for “fatigue”; Tan-Shi-Yu-Zhi was the indicator for “nausea”; Yang-Xu and Yin-Xu were indicators for “frequent urination.” Our findings also indicate that stress level affects constitutional changes and that stress and constitutional change affect the incidence of discomfort. This research can help healthcare professionals observe these discomforts and provide individualized care for pregnant women, to nurture pregnant women into neutral-type constitution, minimize their levels of discomfort, and promote the health of the fetus and the mother.

## 1. Introduction

People typically fault an individual's constitution for physical illness. Is constitution changeable? If so, when will it change? These issues have been widely discussed. Wang [1] notes that a constitution is, in part, genetically determined and, in part, acquired. It is relatively stable in morphology, structure, and function and is associated with personality.

Menstruation, pregnancy, labor, and breastfeeding are female-specific physiological phenomena. From the perspective of traditional Chinese medicine (TCM), for females, “liver” is the fundamental of innate endowment and yin-blood represents the body; “spleen” is the fundamental of acquired constitution and the source of qi, where blood is generated. Therefore, menstruation, pregnancy, labor, and breastfeeding all depend on coordination among the liver, spleen, and kidney. This coordination provides sufficient essences and blood as well as activated yang qi [2]. TCM's constitutional theory states that pregnant women with neutral-type constitution can adapt to physiological and psychological changes well, while women with Spleen-Xu are vulnerable to hypogalactia and pregnancy-related nausea and vomiting, and women with Yang-Xu usually experience edema symptoms during pregnancy. Furthermore, if pregnant women experience anxiety, this may enhance epinephrine secretion, which inhibits bone development in babies [3]. Therefore, understanding the constitution of pregnant women can prevent discomfort and/or complications. Wang et al. [4] published the only study to investigate the relationship between prepregnancy constitution and discomfort in the first trimester. This research aimed to explore the correlations among constitution, stress, and discomfort among women in the first trimester of pregnancy using the Traditional Chinese Medical Constitutional Scale (TCMCS). Our findings elucidate the transformation of pathological constitution to neutral-type constitution, alleviating discomfort during pregnancy, and promoting the health and growth of the fetus. 

### 1.1. Literature Review

From a genetic perspective, constitution represents the characteristics of the individual, which develop gradually, influenced by slow and latent environmental factors during growth, development, and aging. Throughout these processes, constitution remains relatively stable and evolves through variable stages of development. Hence, constitution is not constantly unchangeable; it changes gradually on the basis of genetics, influenced by growth, environment, nutrition, and lifestyle.

Formation of the constitution is greatly influenced by age, environment, and lifestyle. Tian Nian of Ling Shu (Miraculous Pivot) states that “at the age of 20, blood and qi are exuberant; at the age of 30, the five zang-organs localized; at the age of 40, all five zang-organs, six fu-organs, and the 12 regular meridians are merged strongly with great exuberance and interstices but start to decline,” which explains why a body has its own specific physical situations including the exuberance or debilitation of qi, blood, zang-organs, and fu-organs with increasing age [5]. People always stay indoors with air conditioning during hot seasons and with heaters during cold seasons. This may damage normal physiological functions and destroy internal homeostasis. Excessive cigarette smoking and alcohol consumption also facilitate the accumulation of dampness and heat, which enhances the formation of the Dampness-Heat constitution [6]; being over-worked or overrested are additional factors affecting constitution. Jutong Lun (Pain Syndrome) in SuWen (Plain Questions) states that “physical exertion leads to qi desertion,” indicating that overexerting results in consumption of qi and yin and yang blood, weakening the constitution and rendering the individual susceptible to illness [5].

In females, blood is fundamental, and work is accomplished by the qi, especially by the spleen-stomach qi. Sufficient qi and blood are essential for pregnancy and labor [7]. Chen and Zeng [8] have found that Yin-Xu leads to bleeding and mood instability during pregnancy; Yang-Xu results in pitting edema of the legs; Tan-Shi causes edema of the limbs; and Yu-Zhi may suffer from abdominal pain and chest oppression. Qibing Lun (Extraordinary Diseases) in SuWen mentioned that “A baby born with epilepsy, a so-called fetal disease, results from a mother who is frightened during pregnancy, causing her qi to go upward but not downward. This accumulation of essence and qi then leads to epilepsy in fetus” [5, 9]. This means that the constitution and its effects during pregnancy are associated with pregnancy-related discomfort and fetal conditions.

Pregnant women encounter internal and external changes as well as physiological and psychological symptoms due to hormonal changes. Common symptoms of discomfort during the first trimester are nausea, vomiting [4, 10], dizziness [10], frequent urination, fatigue [4], breast tenderness, heavy vaginal discharge, and mood swings [11]. Approximately 50–80% of pregnant women experience nausea and vomiting, which are the two most common symptoms, during the first trimester [12, 13]. The exact mechanism underlying nausea and vomiting in pregnancy remains unknown. In the view of Western medicine, human chorionic gonadotropin (hCG) and progesterone are considered to be the affecting factors. TCM holds that nausea and vomiting may result from the following factors: (1) weakness of the spleen-stomach, (2) malfunction in absorption and excretion, (3) stomach upset resulting from blood that flows into the placenta and stimulates uterine contractions after conception, and (4) blood and qi deficiency as well as poor circulation in weak constitutions due to growth of the placenta [14]. Dizziness is presented to represent unstable vasomotor or postural hypotension resulting from reduced vascular tension and peripheral vascular resistance [11, 15, 16]. TCM defines dizziness as “dizziness in pregnancy” or “gravid vertigo.” When pregnant women's constitutions are not neutral, they are susceptible to dizziness due to hyperactivity of liver yang [17]. Breast tenderness causes a feeling of swelling in the breasts or sensitive nipples due to elevated concentrations of estrogen and progesterone [11, 15, 16]. Frequent urination is attributed to the reduction of bladder volume because of the bladder oppression by the growing uterus [11]. TCM defines frequent urination as “shifted bladder,” meaning dysuria due to pressure of the fetus, which usually occurs in women with Qi-Xu or Kidney-Xu [17]. Pregnant women may experience loss of energy, drowsiness, and fatigue because of elevated basal metabolic rate (BMR), increasing demands on heart and lung circulations, and interactions of the hormone relaxin in ovaries [18, 19]. Nonitching, smelly, or yellow and white vaginal discharge results from lactate, which is produced by glycogenolysis of lactobacillus acidophilus in vaginal epithelial cells and from the proliferation of vaginal mucosa due to increased estrogen [15, 16].

In addition, essence-spirit and mood are external expressions of zang-organs, fu-organs, qi, and blood. Inappropriate essence-spirit and mood (e.g., stress and emotional tension) affect the qi movement of zang-organs and fu-organs and obstruct the qi and blood movement, which will affect the constitution. The emotional effects observed most frequently include joy, anger, anxiety, desire, sorrow, fear, and fright. When one feels stressed, one experiences anxiety and desire. Yin Yang Ying Xiang Da Lun in SuWen points out that “anxiety impairs lungs; desire impairs spleen.” Anxiety causes qi depression, resulting in breathing problems and distension discomfort in chests; desire causes qi stagnation, resulting in poor appetite and fatigue. Ben Cang of Ling Shu states that “mood harmony makes concentrated spirit by which regret and anger will not be aroused, and five zang-organs will not be affected by pathogens” [5]. The seven emotional effects listed above should be coordinated and harmonious, or they will exert negative effects on the constitution. For example, Yu-Xie or Tan-Shi constitution is formed because of excessive and unbalanced emotions [20–22]. Long-term negative emotions will therefore affect the coordination and harmony of qi and blood and may lead to the development of a pathological constitution. Psychological factors affecting pregnant women include motivation of pregnancy, planned pregnancy or not, individual characteristics and maturity, and past labor experience of self and family [11]. Fast and dramatic mood swings, a sense of fragility toward self-image and fetus, and the relationship with spouse are additional factors affecting mood changes [18, 19]. TCM defines “pregnancy vexation” as depression, irritability, worry, or even sleeping disorders occurring during pregnancy [17]. The reason is “heat,” which disturbs heart and lungs. Pregnant women with Yin-Xu, Qi-Xu, and Tan-Shi tend to have extremely insufficient yin-blood, which enhances heat and engenders vexation [23].

**Figure 1:**
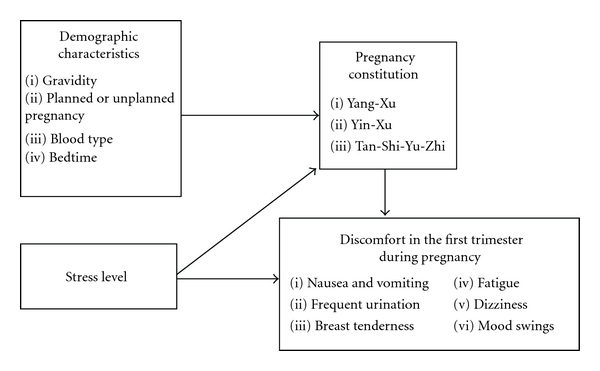
A study framework for correlations among constitution, stress, and discomfort in the first trimester during pregnancy.

In light of the above literature review, we understand that the congenital constitution of the fetus is determined by the maternal constitution and that varied constitutions at different stages formed through the processes of growth, development, and all types of acquired external factors. Aside from the effects on the fetus, constitution during pregnancy causes various symptoms of discomfort for the mother. Therefore, to prevent the occurrence of pregnancy complications and to preserve a healthy and neutral-type constitution in the fetus, we should understand the constitution tendency of pregnant women and accurately adjust their constitution so as to achieve sufficient qi and blood as well as yin-yang balance to meet the needs of mother and fetus. Wang et al. [4] examined the prepregnancy constitution and concluded that “frequent urination,” “fatigue,” “heavy vaginal discharge,” “dizziness,” and “mood swings” are significantly positively correlated with Yin-Xu, Yang-Xu, and Tan-Shi-Yu-Zhi. Nevertheless, little is known about the correlations among constitutions and discomforts during pregnancy. Seeking further understanding and evidence-based data, this research sought to explore the correlations among constitution, stress, and discomfort in the first trimester during pregnancy (Figure 1). 

### 1.2. Research Questions

The research questions of this study were (1) what are the relationships among constitution, stress, and discomfort in the first trimester during pregnancy? and (2) what are the contributions of the constitution and stress to discomfort in the first trimester during pregnancy? 

## 2. Methods

### 2.1. Participants

A descriptive and correlational research design was utilized. Structured questionnaires were used to collect data from a convenience sampling of 261 pregnant women who received prenatal examination from obstetrics and gynecology clinics and district teaching hospitals in southern Taiwan. Inclusion criteria included women aged 21–48, 6–13 gestational weeks, without pregnancy complications and systemic diseases (such as diabetes mellitus, hypertension, systemic lupus erythematosus, and heart disease), Mandarin- or Taiwanese-speaking, and willing to participate in the study. This study was approved by the Institutional Review Board of Kaoshiung Medical University Hospital (KMUH-IRB-960408) and conducted after the informed consents of participants were obtained. All questionnaires were answered anonymously, and each participant had the right to join or drop during the entire study process. 

### 2.2. Research Instruments

In this study, research instruments, which were developed based on literature review and expert validity, included the demographic data sheet, Traditional Chinese Medical Constitutional Scale (TCMCS), Visual Analogue Scale (VAS) on Stress, and Evaluation List of Uncomfortable Symptoms During the First Trimester of Pregnancy. Demographic data include obstetrics data, age, gravidity, planned pregnancy or not, exercise regularity, and bedtime. TCMCS, developed by Su [24], was used to measure the physiological state of pregnancy constitution. It is a 44-item, 5-point Likert scale (from 1 (never happen) to 5 (always happen)) and was composed of three independent constitution scales, including 19 items in Yang-Xu (Yang deficiency; score range 19–95) [25, 26], 19 items in Yin-Xu (Yin deficiency; score range 19–95), and 16 items in Tan-Shi-Yu-Zhi (score range 16–80). Since some items among these three scales were overlapping, TCMCS eventually comprised a total of 44 items (scoe range 44–220). The higher the scores, the more deviation the constitution. The factor analysis of the three independent constitution scales revealed that one factor accounted for 56.4% of the variance in Yang-Xu, 52.3% in Yin-Xu, and 56.9% in Tan-Shi-Yu-Zhi [24]. In the previous study, Cronbach's *α* in each constitution subscale was between  .78 and  .90 [4], while Cronbach's *α* was  .84, .80, and  .83, respectively, in this study. Cronbach's *α* of the TCMCS was  .91. To evaluate the degree of women's perceived stress during pregnancy, we adopted a 10 cm vertical Visual Analogue Scale (VAS) with anchors at each end: “0, no stress” on the bottom and “10, the worst stress” on the top; higher scores indicate greater perceived stress. The VAS is a reliable and valid measurement tool that was wildly used [27]. We used the Evaluation List of Uncomfortable Symptoms During the First Trimester of Pregnancy, a checklist based on the 4-point Likert scale (0 = none; 1 = mild; 2 = moderate; 3 = severe; 4 = extremely severe), to assess the severity of discomfort for pregnant women, including nausea, vomiting, breast tenderness, frequent urination, fatigue, and mood swings. The content validity index (CVI) and Cronbach's *α* of the scale of uncomfortable symptoms were  .72 and  .72 respectively, while the value reported by a previous study was  .64 [4]. 

### 2.3. Data Analysis

The collected data were analyzed using SPSS 12.0 statistical software. Descriptive statistics were used to describe demographic characteristics. Inferential statistical methods, such as one-way ANOVA, *t*-test, and Pearson's product-moment correlation, were used to analyze the differences and the correlations among variables. Multinomial logistic regression was used to predict the indicators of discomfort. 

## 3. Results

Participants in this study were aged between 21 and 42, with a mean age of 29.8 ± 3.96; gestational weeks ranged from 6 to 13, with a mean week of 9.16 ± 2.22. Most of the women had no experience of miscarriage (*n* = 196, 75.1%), and most were employed outside the home (*n* = 190, 72.8%). Educational levels were university and junior college (*n* = 90, 34.5%; and *n* = 77, 29.5%, resp.) with a mean of 13.06 ± 1.03 years of education. The majority of participants had no habits of smoking and drinking (*n* = 251, 96.2%); body mass index (BMI) ranged from 15.8 to 37.8, with a mean BMI of 21.41 ± 3.31; stress level during pregnancy ranged from score 0 to10, with a mean score of 4.41 ± 2.77 (see Table 1).

 Participants in this study scored between 20 and 56 (*M* = 31.09 ± 6.75) in Yin-Xu, 20–69 (*M* = 34.94 ± 8.59) in Yang-Xu, and 16–54 (*M* = 28.33 ± 6.67) in Tan-Shi-Yu-Zhi. The top five common symptoms of discomfort in the first trimester pregnancy (within 13 weeks) were fatigue (*n* = 257, 98.5%), nausea (*n* = 232, 88.9%), frequent urination (*n* = 230, 88.1%), breast tenderness (*n* = 224, 85.8%), and heavy vaginal discharge (*n* = 220, 84.3%). There were 17 participants (7.2%) who reported other symptoms, including abdominal oppressive pain, headache, bleeding, chest oppression, lumbar soreness, dyspnea, taste changes, stomach distention, sleeping disorder, and impaired vision. In terms of severity level, most patients reported “moderate” fatigue (*n* = 110, 42.1%) and frequent urination (*n* = 109, 41.8%), “mild” nausea (*n* = 110, 42.1%), breast tenderness (*n* = 122, 46.7%), and heavy vaginal discharge (*n* = 129, 49.4%). Total scores of discomfort ranged from 0 to 24, with a mean score of 11.5 ± 4.6. Each woman might have 0 to 10 symptoms of discomfort, with a mean of 6.98.

Pearson's product-moment correlation indicated that stress has significantly positive correlations with Yang-Xu (*r* = .27, *P* < .001), Yin-Xu (*r* = .20, *P* < .001), and Tan-Shi-Yu-Zhi (*r* = .27, *P* < .001). Yin-Xu and Tan-Shi-Yu-Zhi have significant correlations with all symptoms of discomfort in the first trimester, while Yang-Xu has significant correlations with all symptoms of discomfort except for “runny nose.” We further examined the correlations between total scores of discomfort and pregnancy constitution and discovered that Yin-Xu (*r* = .51, *P* < .001), Yang-Xu (*r* = .54, *P* < .001), and Tan-Shi-Yu-Zhi (*r* = .59, *P* < .001) were significantly positively correlated with all symptoms of discomfort. Constitution during pregnancy was also significantly positively correlated with the extent of discomfort (see Table 2).

Based on the above findings, the symptoms of discomfort in the first trimester were correlated with “Yin-Xu score,” “Yang-Xu score,” “Tan-Shi-Yu-Zhi score,” and “stress level.” By using multinomial logistic regression, the significant indicators for the top five symptoms of discomfort—“nausea,” “vomiting,” “frequent urination,” “breast tenderness,” and “heavy vaginal discharge”—were determined. We integrated the variable of stress level (“extremely severe” to “severe”) due to the small sample size and set “none” as the comparison group, performing the same integration for patients who reported “none” or “mild” fatigue, with patients who reported “mild” fatigue serving as the comparison group. With demographic data such as age, years of education, number of miscarriages, BMI, bedtime and blood type under control, the significant factors for symptoms of discomfort were “Yin-Xu score,” “Yang-Xu score,” “Tan-Shi-Yu-Zhi score,” and “stress level” (see Table 3). Further results, using estimator B, showed “Tan-Shi-Yu-Zhi” and “stress” were the two indicators for “fatigue.” The odds ratio (OR) of moderate and severe fatigue was 1.18 times and 1.30 times, respectively, higher than that of mild fatigue. The OR value of severe fatigue was 1.23 times higher than that of mild fatigue. We also found “Tan-Shi-Yu-Zhi” was the indicator for “nausea”; “Yang-Xu” and “Yin-Xu” were the two indicators for “frequent urination”; and “Yang-Xu” was the indicator for “heavy vaginal discharge.” No significant result was observed for the other symptoms (see Table 4). 

## 4. Discussion

The findings of this study are the very first understanding of the relationships among constitution, stress, and discomfort symptoms for pregnant women in the first trimester. We found that the significant indicators for the top five symptoms of discomfort include “stress level,” “Yang-Xu score,” “Yin-Xu score,” and “Tan-Shi-Yu-Zhi score.” The results further indicate that “Tan-Shi-Yu-Zhi” and “stress” were the two indicators for fatigue, “Tan-Shi-Yu-Zhi” was the indicator for nausea, “Yang-Xu” and “Yin-Xu” were the two indicators for frequent urination; and “Yang-Xu” was the indicator for heavy vaginal discharge. 

This research also suggests that the increase in frequent urination, fatigue, and vaginal discharge has a significantly positive correlation with Yang-Xu and Yin-Xu, which is consistent with the results of Lo and Lo [17] and Pan et al. [28] showing that pregnant women whose Yin-Xu constitution cannot control “heart fire”, tend to have frequent urination [28]. In addition, the bladders of pregnant woman are compressed by the growing uterus, leading to dysuria and reduced bladder volume if they have Qi-Xu or Kidney-Xu constitutions [17]. 

Our findings suggest that fatigue has significant and positive correlation with the constitution of Yang-Xu, Yin-Xu, and Tan-Shi-Yu-Zhi, which is consistent with the view of Chen and Zeng [8] and Miao [29]. In the study of female constitution categorization, Chen and Zeng [8] claim that women with Qi-Xu tend to experience fatigue, lack of strength, and less communication, while those with Tan-Shi-Yu-Zhi tend to experience somnolence and less movement; Miao [29] also finds fatigue and lack of strength in the Qi-Xu constitution. We also confirm the statement of Li and Hsu [30] that blood is the source of qi; qi is dependent on essence and carried by fluids. Obstructing fluid and humor leads to stagnant qi and indirectly affects the formation of blood and finally cause insufficiency in sources of qi and yang, which will cause fatigue and exhaustion. Our findings reveal that heavy vaginal discharge has a significantly positive correlation with Yang-Xu, Yin-Xu, and Tan-Shi-Yu-Zhi. Heavy vaginal discharge is associated with estrogen; however, the human endocrine system is discussed rarely in the TCM literature, so relevant findings in this study cannot be verified or further discussed. Nevertheless, our findings indicate that Tan-Shi-Yu-Zhi is susceptible to heavy vaginal discharge, which supports indirectly the statement of TCM. That is, shi (dampness) is considered a yin pathogen that will accumulate in zang-organs, fu-organs, and regular meridians, obstruct qi movement, and damage yang qi; shi is characterized by its heaviness and stickiness, such as sticky urination and defecation, heavy and sticky vaginal discharge, and greasy tongue fur [30].

Our research has shown that there are two emotion-related symptoms, “poor appetite due to stress during pregnancy” and “stress levels”, significantly and positively correlated with Yang-Xu, Yin-Xu, and Tan-Shi-Yu-Zhi; the higher the stress level, the greater the tendency to these three constitutions. The results are in agreement with many previous statements. For example, TCM believes qi and blood are the foundation of essence-spirit and mood, so disharmonious essence-spirit and mood will disturb qi movement in zang-organs and fu-organs and might cause “six depressions”: stagnation of qi, blood, damp, fire, phlegm, and food [30, 31]. Yang [22] and Li [21] report that disharmonious mood causes qi and blood disturbance, which may be further related to the formation of Yu-Xie and Tan-Shi. In sum, stressed emotions will cause the insufficiency and disturbance of qi, blood, and yin-yang in zang-organs and fu-organs, resulting in negative effects on constitution.

Our findings indicate that the top five symptoms of discomfort during the first trimester of pregnancy are fatigue, nausea, frequent urination, breast tenderness, and heavy vaginal discharge, which are partially consistent with the findings of Cheng [32]. Cheng has shown that fatigue, nausea, hunger, sense of happiness, and nipple/breast tenderness are the top five psychosomatic symptoms during the first trimester among normal pregnant women. There are slightly differences between these two studies. In our study, the top five symptoms of discomfort were focused only on discomfort symptoms, while Cheng's study included positive emotion. This study presents that 98.3% of the participants feel fatigue, which is similar to the findings (approximately 90%) reported by Rodriguez et al. [33] and Chou et al. [34]. This study shows that the incidence of nausea and vomiting is 88.09% and 65.11% respectively, which is similar to the findings (50–80%) of Koren and Maltepe [12] and of Koch et al. [13]. 

## 5. Conclusions

This research examines the correlations among constitution, demographic characteristics, stress, and discomfort experienced by women during their first trimester of pregnancy. The findings of this study suggest that stress is significantly positively correlated with Yang-Xu, Yin-Xu, and Tan-Shi-Yu-Zhi; total scores and the number of discomfort symptoms during pregnancy are significantly positively correlated with pregnancy constitution. Among those, “frequent urination,” “fatigue,” “heavy vaginal discharge,” “nausea,” “vomiting”, “mood swings,” “nasal congestion,” “dizziness,” and “breast tenderness” are significantly positively correlated with Yin-Xu, Yang-Xu, and Tan-Shi-Yu-Zhi; “running nose” is significantly positively correlated with Yin-Xu and Tan-Shi-Yu-Zhi. These findings indicate that constitution and discomfort symptoms during the first trimester of pregnancy are closely related. This research supports the effect of stress levels on constitutional changes and indicates that appropriate emotional management and regular and healthy bedtime schedules are required to reduce the development of Xu constitution during pregnancy. Healthcare professionals should not only observe the discomfort symptoms of pregnant women but also provide individualized care at the proper time to help pregnant women develop a neutral-type constitution to enhance their comfort during pregnancy and promote the health of fetus and mother.

In addition, the majority of Taiwanese believe that TCM principles are more natural; however, the perinatal care typically provided is dictated by the tenets of Western medicine. The use of the TCM constitutional scale can help pregnant women understand their own constitution and can assist healthcare professionals in providing individualized care and dietary education. The limitation of this cross-sectional research is that it only examines the pregnant women recruited from southern Taiwan. Prenatal and postnatal constitutions of women may be changeable. However, the north-south regional disparity may be a key factor in these constitution issues. Hence, future research could adopt a longitudinal study to examine women's constitution during the perinatal period among patients from the north, central, and south regions of Taiwan so as to obtain more comprehensive evidence-based information. Another limitation is that we only make the number of primigravida or multigravida among 261 pregnant women clear, but not include the number of primipara or multipara. Since the childbirth may be also influenced to the formation of constitution, future research should include the number of primipara or multipara to provide more suitable evidence-based data. 

## Figures and Tables

**Table 1:** Demographic characteristics of participants (*N* = 261).

Item	*n*	%	Item	*n*	%
Gravidity			Workload		
Primigravida	129	49.4	Mild	92	35.2
Multigravida	132	50.5	Medium or Excessive	169	64.8
			Planned pregnancy		
Blood type			No	140	53.6
A	59	22.6	Yes	121	46.1
B	71	27.2	Poor appetite due to stress during pregnancy		
O	113	43.3	No	178	68.2
AB	18	6.9	Yes	83	31.8
Exercises regularly			Bedtime during pregnancy		
No	204	78.2	Before 11PM	131	50.2
Yes	57	21.8	After 11PM	130	49.8

**Table 2:** Correlations among symptoms of discomfort in the first trimester, constitution, and stress.

Item	Yang-Xu	Yin-Xu	Tan-Shi-Yu-Zhi	Stress
(1) Nausea	.32**	.30**	.36**	.18**
(2) Vomiting	.22**	.26**	.28**	.11
(3) Breast tenderness	.18**	.14*	.15**	.06
(4) Frequent urination	.23**	.26**	.24**	.03
(5) Fatigue	.43**	.35**	.44**	.29**
(6) Heavy vaginal discharge	.31**	.27**	.32**	.15*
(7) Nasal congestion	.21**	.23**	.24**	.05
(8) Running nose	.10	.14*	.17**	.03
(9) Dizziness	.39**	.38**	.41**	.30**
(10) Mood swings	.44**	.37**	.47**	.36**
Total score of discomfort	.54**	.51**	.59**	.30**
Number of discomfort	.36**	.41**	.44**	.21**

*P < .05   **P < .01

**Table 3:** Analysis of multinomial logistic regression for the significant indicators of various symptoms of discomfort (*N* = 261).

Symptoms of discomfort	Significant indicators		
	indicator	degrees of freedom	*P*-value
Fatigue	Tan-Shi-Yu-Zhi score	2	<.001
	Stress	2	.013
			
Nausea	Tan-Shi-Yu-Zhi score	3	<.001
			
Frequent urination	Yang-Xu score	3	.009
	Yin-Xu score	3	.002
			
Breast tenderness	Yang-Xu score	3	.025
			
Heavy vaginal discharge	Yang-Xu score	2	<.001

*Note* 1. Control variables: blood type, age, gestational weeks, gravidity, times of miscarriage, years of education, BMI, workload, exercise habits, planned pregnancy, stress due to pregnancy, and bedtime during pregnancy.

*Note* 2. Major indicators of this study: Yin-Xu, Yang-Xu, Tan-Shi-Yu-Zhi, and stress.

**Table 4:** Multinomial logistic regression of effects of significant indicators on symptoms of discomfort (*N* = 261).

Discomfort (outcome variable)	Explanatory variable	Degrees of freedom	*P*-value	OR	95% CI
*Fatigue*	Tan-Shi-Yu-Zhi				
Moderate versus mild		1	<.001	1.18	(1.10, 1.27)
Severe versus mild		1	<.001	1.30	(1.20, 1.41)
	Stress				
Moderate versus mild		1	.17	1.09	(.96, 1.23)
Severe versus mild		1	<.001	1.23	(1.07, 1.42)
*Nausea*	Tan-Shi-Yu-Zhi				
Mild versus none		1	.82	1.01	(.93, 1.09)
Moderate versus none		1	<.001	1.16	(1.06, 1.26)
Severe versus none		1	<.001	1.16	(1.06, 1.27)
*Frequent urination*	Yang-Xu				
Mild versus none		1	<.001	.88	(.82, .96)
Moderate versus none		1	.34	.92	(.85, .96)
Severe versus none		1	.47	.97	(.88, 1.06)
	Yin-Xu				
Mild versus none		1	<.001	1.20	(1.06, 1.34)
Moderate versus none		1	<.001	1.24	(1.10, 1.39)
Severe versus none		1	.02	1.18	(1.03, 1.36)
*Breast tenderness*	Yang-Xu				
Mild versus none		1	.48	.98	(.94, 1.03)
Moderate versus none		1	.27	1.03	(.98, 1.08)
Severe versus none		1	.14	1.04	(.99, 1.11)
*Heavy vaginal discharge*	Yang-Xu				
Mild versus none		1	.06	1.05	(.99, 1.11)
Moderate versus none		1	<.001	1.12	(1.06, 1.18)
